# Multilevel associations between skills use, engagement, and treatment outcome in self-guided internet-delivered dialectical behavior therapy for substance use disorders

**DOI:** 10.1371/journal.pmen.0000564

**Published:** 2026-03-24

**Authors:** Danielle Downie, Alexander R. Daros, Chelsey R. Wilks, Lena C. Quilty

**Affiliations:** 1 Department of Psychological Clinical Science, University of Toronto, Toronto, Ontario, Canada; 2 Campbell Family Mental Health Research Institute, Centre for Addiction and Mental Health, Toronto, Ontario, Canada; 3 Department of Psychology, University of Windsor, Windsor, Ontario, Canada; 4 Tori Health, Los Angeles, California, United States of America; 5 Department of Psychiatry, University of Toronto, Ontario, Canada; Instituto Federal do Maranhão: Instituto Federal de Educacao Ciencia e Tecnologia do Maranhão, BRAZIL

## Abstract

Internet-delivered dialectical behavior therapy (iDBT) represents a scalable and accessible treatment approach for individuals with substance use disorders (SUDs), yet little is known about the mechanisms of change and the role of engagement in this format. This study examined within- and between-person associations between changes in skills-based constructs (mindfulness, DBT skills use, emotion dysregulation) and two treatment outcomes (SUD severity and functional disability) across a 12-week self-guided iDBT program. The moderating role of treatment engagement was also evaluated. 72 participants with past year SUDs were randomized to immediate or delayed iDBT and completed assessments at baseline and weeks 4, 8, and 12. Multilevel models were used to assess within- and between-person associations between skills and outcomes over time. Several indices of engagement were used including treatment acceptability, usability, and hours/days spent on iDBT. Within-person increases in emotion dysregulation were associated with higher SUD severity and disability, over and above respective between-person effects. DBT skills and mindfulness were not significantly associated with outcomes after correction for multiple tests, though the latter produced a small-medium effect size at the between-persons level. There were no significant moderation effects of treatment outcomes after correction for multiple tests, but several produced small-medium effect sizes. These preliminary findings support the role of emotion dysregulation as a key correlate of treatment response and promote further research within an iDBT context. Future work should refine measurement of DBT skill acquisition and investigate longer-term functional impacts of digital DBT interventions.

## Introduction

Substance use disorders (SUDs), which involve the problematic use of alcohol, nicotine, cannabis, and other substances, are a pervasive global health concern with severe impacts on individuals, families, and society at large [1]. SUDs are associated with a high degree of functional disability, including impaired relationships, employment, and overall functioning [2]. Broad and scalable intervention efforts are critical for reducing long-term harms. International institutions emphasize the need for equitable access to treatment and harm reduction services to combat this ongoing public health issue [3,4]. However, research suggests that fewer than 10% of people with SUDs receive specialized treatment, and of those who receive care; dropout rates often exceed 30% for psychological interventions [5]. These challenges highlight the necessity of improving access to treatment as well as engagement with them, to ensure interventions meet the diverse needs of individuals with SUD.

There is a long-standing recognition that substance use functions to regulate emotions [6]. This regulatory function leads to reinforcement and maintenance of substance use [7]. Given the prominent role of emotion regulation in mental disorders often co-occur with harmful substance use, such as mood and anxiety disorders [8], psychological interventions that reduce harmful substance use by developing a range of healthy emotion regulation skills represent promising transdiagnostic treatment approaches that could improve not only SUDs but also conditions that often co-occur with SUDs. Dialectical behavior therapy (DBT) was initially developed for the treatment of individuals with severe suicide risk and individuals with borderline personality disorder (BPD) [9,10]. Due to the high number of individuals with BPD presenting with co-occurring SUDs, specialized DBT content was created to target and address harmful substance use [11]. DBT for individuals with SUDs addresses intense emotions (e.g., craving, withdrawal), impulsive behaviors (e.g., managing urges, avoiding cues), and interpersonal effectiveness (e.g., increasing community involvement) using a skills-based approach. The goal of DBT for SUDs is to reduce harmful substance use and dysfunctional behaviours while increasing behavioural control [11,12].

Research supports the feasibility and efficacy of DBT for SUDs, wherein DBT includes the fulsome offering of in-person group and individual therapy sessions, along with phone coaching in crisis situations and consultation for therapists. This comprehensive DBT protocol is associated with decreased frequency of substance use, improved social functioning, and enhanced emotion regulation in early trials in those with SUDs with or without co-occurring BPD [13–17]. Additional research has supported the use of stand-alone group-delivered DBT skills training (often abbreviated as DBT-ST) for SUDs [18–20]. One recent systematic review of DBT-ST for SUDs reported reduced substance use and improved emotion regulation within participants over treatment [21]. Collectively, results support both comprehensive DBT and skills training formats to achieve positive outcomes for individuals with SUDs. To further increase the reach of DBT, researchers have developed mobile and internet-delivered formats to study its impact on SUDs using a scalable format that increases accessibility. One recent systematic review of DBT-ST for SUDs reported reduced substance use and improved emotion regulation within participants over treatment [21]. Collectively, results support both comprehensive DBT and skills training formats to achieve positive outcomes for individuals with SUDs. To further increase the reach of DBT, researchers have developed mobile and internet-delivered formats to study its impact on SUDs using a scalable format that increases accessibility.

Mobile and internet-delivered DBT (often abbreviated as iDBT) offers an increasingly accessible and flexible approach to care. This delivery method has gained traction over the past decade due to high demands for mental health treatment (compounded by the COVID-19 pandemic) and the challenges in attending in-person treatments such as comprehensive DBT [22,23]. The first randomized controlled trial of self-guided iDBT for harmful substance use recruited individuals with high suicidality and heavy alcohol use [24]. Individuals were randomized to immediate or delayed access, with roughly 85% of participants completing the first module and one-third completing all eight. Those who remained in the study longer and completed more modules reported significantly reduced suicidal ideation, alcohol quantity and severity, and emotion dysregulation over 16 weeks. Schroeder and colleagues [25] demonstrated feasibility and acceptability for the first iDBT mobile app, called *Pocket Skills,* as an adjunct to standard DBT treatment in a non-randomized trial of participants with mostly depressive and anxiety disorders. The sample consisted of 73 individuals with participants meeting criteria for depression (n = 38), generalized anxiety disorder (n = 35), BPD (n = 29), post-traumatic stress disorder (n = 20), bipolar disorder (n = 10), and other disorders. After 4 weeks in the study, the authors reported decreased depression and anxiety symptoms, while also increasing DBT skills use. Results suggested that *Pocket Skills* helped participants engage in their DBT and practice and implement skills in their environmental context due to its mobile nature.

*Pocket Skills* was revised and investigated as a self-guided intervention for SUDs in a subsequent randomized controlled trial [26]. Overall, participants reported reductions in both substance dependence and functional disability, supporting the potential of iDBT in treating SUDs in a complex sample with a high degree of concurrent mental health disorders. Nevertheless, as with many treatments for SUDs, there was variability in the severity of baseline symptom severity and treatment outcome. Therefore, the present manuscript focuses specifically on secondary analyses examining the between- and within-person associations between three key targets of DBT and treatment outcome, as well as potential moderation of engagement.

Research on the potential mechanisms underlying DBT outcomes has typically focused on gains in mindfulness, emotion regulation ability, distress tolerance, and DBT skills [27]. Increases in DBT skills use were associated with improved treatment outcomes in a transdiagnostic DBT group treatment, suggesting that skills use may be a mediator of treatment outcomes in DBT more broadly [28,29]. In a sample of adolescents undergoing DBT, both improvements in emotion regulation and interpersonal sensitivity were significant predictors of change in depression and anxiety [30]. Participants in this sample included youth aged 14–25 who had a history of suicide attempts in the last 5 years. Additional research supports improvements in emotion regulation and DBT skills as mediator of suicidality and self-harm reductions in youth undergoing DBT for suicidal behaviors and self-harm [31]. Given that individuals will vary in their mindfulness, emotion regulation, and DBT skills at the outset of an intervention, and have different improvement trajectories, it is important to consider the between- and within-person variation of these variables over the course of treatment.

Engagement in treatment is also critical to clinical outcomes, and yet, it remains an ill-defined construct. In traditional psychotherapy, engagement can be measured as attendance and participation in treatment, as well as homework completion, and is often associated with improved outcomes [32,33]. Within a standard DBT context, increased weekly homework completion and participation in phone coaching predicted improved suicidal behaviors and substance use in an outpatient program [34]. With the proliferation of digital health interventions, there have been attempts to conceptualize and define engagement given its association with treatment outcome [35,36]. Rather than focusing only on behavioral engagement metrics (e.g., frequency of use and time spent on the intervention), Kelder and colleagues [35] propose cognitive components as well, such as perceptions about the intervention (e.g., its acceptability and usability). In one study of patients with SUDs, the number of modules completed within an online self-guided cognitive behavioral therapy treatment program (e.g., behavioral engagement), was positively associated with reduced substance use [37]. Within the limited iDBT literature, only one study examined the relationship between behavioural engagement and treatment outcomes. However, against expectations, neither module completion nor diary card entries within an iDBT app were predictive of disordered eating symptom improvement [38]. This literature gap provides the opportunity to further understand the relationship between online iDBT treatment engagement and treatment outcomes in SUDs.

## Aims of the present study

The current study sought to extend the results of a previous trial by examining between- and within-person associations for several predictors of treatment outcomes (i.e., SUD severity and functional disability) in individuals with SUDs who received iDBT. Considering the literature on both traditional and internet-delivered DBT formats, we examined whether changes in DBT skills, mindfulness, and emotion dysregulation predicted response to treatment for individuals with SUDs. Using multilevel modelling, we considered both within- and between-person associations between skills-based variables and treatment outcomes, while also considering key demographic and control covariates. We then further examined the moderation of iDBT treatment outcomes by examining several indices of cognitive and behavioural engagement: treatment acceptability, usability, and time spent on the intervention (in hours and unique days accessed). We hypothesized that (1a) increased within-person in emotion dysregulation would be associated with higher SUD severity (i.e., impeding treatment), and (1b) increased within-person mindfulness and DBT skills would be associated with lower SUD severity, over and above between-person effects. Next, we hypothesized (2a) that we would find evidence of moderation for time-varying, cognitive engagement variables (e.g., treatment acceptability and usability) at the within-person level and (2b) behavioural engagement variables (e.g., hours/days spent on iDBT) at the between-person level, such that higher engagement would strengthen the relationships between time and SUD severity outcome. Finally, we hypothesized that (3) we would replicate the SUD severity findings using a second treatment outcome, functional disability. We also considered potential issues related to normality by adding sensitivity analyses using a generalized linear mixed model approach.

## Methods

### Ethics statement

Ethics approval was granted by the Centre for Addiction and Mental Health to the principal investigator (LCQ). All interested participants were provided an informed consent form prior to joining the study. The informed consent form was reviewed with the participant by a research team member and written consent was received prior to participation in the study.

### Study design and recruitment

This study is a secondary analysis of a preregistered, two-arm, single-blinded, parallel-group randomized controlled trial of iDBT for SUDs (NCT05094440; [26]). The research questions and analyses presented in the current study are unique and do not overlap with previous work. Participants were divided into two groups: those receiving immediate access to iDBT and those assigned to a waitlist for four weeks before receiving access. Participant recruitment for the current study occurred after a piloting period following initial Research Ethics Board approval #16/2021 (July 12, 2021) at the Centre for Addiction and Mental Health. Following revisions, recruitment for the present study ran from July 20, 2022, to March 31, 2023, and utilized referrals from psychiatric hospital clinicians, waitlists, and research registries, as well as self-referrals from local community advertisements on hospital websites, social media, private clinics, and community organizations. Advertisements targeted individuals seeking to reduce alcohol or substance use, through an internet-delivered intervention provided at no cost. Prospective participants were informed about the study and underwent phone-based pre-screening for eligibility. To be included in the study, participants had to be between the ages of 18–65 years; fluent in English; willing to complete study requirements; not currently enrolled in CBT or DBT (peer support and psychiatric services were permitted); a past year SUD; internet access and literacy; and at least a score of 4 on a modified Contemplation Ladder question (indicating moderate readiness to reduce substance use). Exclusion criteria included practical barriers to participation (e.g., extended absences); acute psychiatric conditions (e.g., suicidality, psychotic disorder) or medical conditions requiring immediate attention; and enrollment in other psychological intervention studies. Use of psychotropic medications did not disqualify participation.

### Procedure

Eligible participants attended a 45-minute baseline session via videoconference and provided electronic informed consent with digital signature to participate in the study. After this, they completed a demographic survey and participated in a semi-structured diagnostic interview. Participants were then randomized into immediate or delayed iDBT groups. Participants in the immediate group received the iDBT website URL and an access code, completing the sign-in process during the session. The delayed access group completed the sign-in process after four weeks in a second, brief videoconference session. Both groups were instructed to spend at least 1–2 hours per week over the first 4 weeks, which was defined as the acute treatment period. Engagement was captured up to 12 weeks post-baseline for both groups.

Questionnaire assessments were conducted at baseline and then 4-, 8-, and 12-weeks post-baseline, using REDCap (Research Electronic Data Capture) [39] and distributed via email or text every four weeks. Automated reminders were sent daily for up to four days, beginning two days before the due date. Additional calls or meetings with the experimenter were offered on an as needed basis for technological support. Participants were compensated an average CAD $59 (maximum CAD $70) for baseline and follow-up assessment completion.

### iDBT intervention

*Pocket Skills 2.0* is an iDBT intervention developed by the third author in collaboration with Microsoft Research and Marsha Linehan, the creator of DBT. It was built upon the most recent DBT manual available [40] and uses a web-based portal built on the Microsoft Azure platform that is compatible with any internet browser. This iDBT intervention incorporates lessons following the core modules of DBT (mindfulness, emotion regulation, distress tolerance, and interpersonal effectiveness) as well as a specific module focused on addiction. Within each module, participants select specific skills and are presented with a brief video featuring Dr. Linehan introducing the skill and its uses. A practice session then ensues with the rule-based chatbot, which allows for feedback through both open-ended text input and a closed selection of responses. The chatbot guides users on how to select skills to use in different situations that may arise as well as the ability to gain points and unlock additional content, which increases user engagement.

### Measures

Demographic information was collected during a brief clinical interview at the baseline meeting. The Diagnostic Assessment and Research Tool (DART; [41]) is a brief semi-structured diagnostic interview that was used to assess depressive, anxiety, bipolar, obsessive-compulsive, trauma and stressor, psychotic, and SUDs according to the *Diagnostic and Statistical Manual of Mental Disorders, Fifth edition* [42]. All interviews were completed by the second author, who is a licensed clinical psychologist. Rates of current mental disorders were as follows: Major Depressive Disorder (51.4%), Persistent Depressive Disorder (25.0%), Bipolar I/II Disorder (8.3%), Panic Disorder (5.6%), Agoraphobia (8.3%), Specific Phobia (4.2%), Generalized Anxiety Disorder (51.4%), Social Anxiety Disorder (30.6%), Post-Traumatic Stress Disorder (25.0%), Alcohol Use Disorder (65.3%), Cannabis Use Disorder (33.3%), Nicotine Use Disorder (20.8%), and Stimulant Use Disorder (12.5%).

### Skills-based and outcome measures

Reliability coefficients for these measures are listed in Table 1.

**Table 1:** Baseline Descriptive Statistics and Between- and Within-Persons Correlations for Demographic and Variables of Interest.

Variables	Mean	SD	Range	Between-person ω	Within-person ω	1	2	3	4	5	6	7	8	9
1. Age	34.89	11.68	19-64	--	--	--								
2. Sex (1 = female, 0 = male)^1^	.66	.47	0-1	--	--	-.12	--							
3. Race (1 = White, 0 = Other Race)^1^	.60	.49	0-1	--	--	.33**	.06	--						
4. Treatment Arm (1 = Immediate, 2 = Delayed)^1^	1.48	.50	1-2	--	--	.01	.05	.04	--					
5. SUD Severity	7.81	3.59	0-15	.95	.76	.36**	.22	-.14	-.15	--	.36**	-.10	.32**	-.14
6. Functional Disability	16.04	9.02	2-43	.94	.78	.07	.21	-.04	-.06	.45**	--	-.17*	.42**	-.33**
7. DBT Skills	1.78	.41	.87-2.74	.94	.80	-.05	.01	.03	.01	-.11	-.21	--	-.15*	-.004
8. Emotion Dysregulation	51.19	12.71	26-75	.98	.81	-.02	.06	-.01	.03	.40**	.59**	-.20	--	-.52**
9. Mindfulness Skills	3.56	.95	1.80-5.67	.94	.85	-.05	-.14	-.26*	-.03	-.37**	-.50**	.21	-.69**	--

*Note*: * *p* < .05; ** p < .01. SD = Standard deviation. SUD = Substance use disorder. dbt = Dialectical behavior therapy. Descriptive statistics (mean, SD, and range) calculated at baseline between-persons. ω = McDonalds omega reliability statistic with between- and within-person estimates provided. Between-person correlations are below the diagonal and use each participant’s mean responses for each measure (or demographic and clinical variables) when conducting the correlations. Within-person correlations are above the diagonal. ^1^Correlations for these variables were assessed using Spearman’s (versus Pearson’s) test.

The *Mindful Attention and Awareness Scale* [43] is a 15-item measure of mindful attention and awareness, which is a primary target of DBT skills training. Items were rated on a 6-point Likert scale (1 = Almost Never to 6 = Almost Always). Higher scores reflect higher levels of dispositional mindfulness ability.

The *Difficulties in Emotion Regulation Scale* [44] is an 18-item self-report measure of difficulties in regulating emotions, including six subscales: emotional awareness, emotional clarity, difficulties in goal orientation, difficulties in impulsivity, nonacceptance, and difficulties selecting strategies. Items are rated on a 5-point Likert scale (1 = Almost Never to 5 = Almost Always). Higher scores indicate greater problems in emotion regulation, and lower scores fewer difficulties managing emotions.

The *DBT Ways of Coping Checklist* [45] is a 59-item self-report measure that assesses the frequency of maladaptive and adaptive skills used to manage difficult situations over the past month, with good internal consistency and test-retest reliability. In this study, we used the 38-item adaptive skills subscale, which includes skillful behaviors often learned in DBT without using DBT-specific language. Items were rated on a 4-point Likert scale (0 = Never Used to 3 = Regularly Used) and higher scores reflect more adaptive skills usage.

The *Severity of Dependence Scale* [46] is a 5-item measure of SUD severity that is well-validated against substance use disorders. Items are rated on a 4-point Likert scale (0 = Never/Almost never to 3 = Always/Nearly always), with higher scores indicating a higher level of substance dependence symptoms. Participants were first asked to indicate which class of substance (including alcohol) they were experiencing the most difficulties with.

The *World Health Organization Disability Assessment Schedule* (Short Form 2.0; [47]) is a 12-item measure assessing six domains related to quality of life: physical health, psychological, social relationships and environment. Items were rated on a 5-point Likert scale (0 = None to 4 = Extreme or cannot do) with higher scores indicating greater functional disability.

### Engagement measures

The *Treatment Acceptability Questionnaire* [48] is a 6-item self-report scale that was administered during follow-up assessments to examine perceived acceptability, safety, and trustworthiness using a 7-point Likert scale from 1 to 7 (anchors differ per item). A total score is created between 7 and 42, with higher scores indicating greater acceptability. In the current study, the between/within ω reliability was.92/.55.

The *mHealth App Usability Questionnaire* [49] is an 18-item scale that was administered during follow-up assessments to examine perceived ease of use, interface and satisfaction, and usefulness of the iDBT platform (between/within ω reliability = .94/.67). Items are rated on a 7-point Likert scale from 1 (strongly agree) to 7 (strongly disagree) and an average score can be used, with lower scores suggesting better usability ratings.

Other measures of engagement included the total amount of time spent on the application (in hours) as well as the number of unique days where participants recorded at least 5 minutes of activity. These measures were calculated based on the duration of the trial (i.e., 12 weeks).

### Statistical analyses

We first collapsed data across treatment arms given that there were no significant group differences in our treatment outcomes of interest (see [26]). Instead, treatment arm was added as a covariate in all analyses. Data were organized as multiple observations nested within individual participants. A total of 261 surveys were attempted across the trial, with no data missing from the baseline. Participants returned at least partially completed follow-up questionnaires at rates of 94% (week 4), 78% (week 8), and 81% (week 12). At follow-up, scores for outcome measures were only used if there were <10% of items missing, and we treated outcome measures with no data as missing. We first examined descriptive statistics for each variable at baseline and used person-level means to examine between-person correlations. For time-varying measures, we also calculated within-person correlations using the *rmcorr* package [50] (Version 0.7) in R, version 4.5.2 (Released 2025-10-31). This package uses a modified ANCOVA framework with subjects as a between-person factor to convert the ratio of the sums of squares of within-person variability of each measure to a Pearson correlation. To examine internal consistencies, we calculated between- and within-person omega reliability [51] statistics using the omegaSEM function from the *MultilevelTools* package (Version 0.2.1).

To test multilevel associations between time-varying predictor variables (e.g., mindfulness, DBT skills, and emotion dysregulation) and treatment outcomes (e.g., SUD severity and functional disability), we first disaggregated their between- and within-person variability [52]. Within-person variables were calculated by subtracting the person-level mean (i.e., person mean centering) from each individual data point. Between-person variables were calculated by subtracting within-person scores from grand-mean and were entered to control for between-person variability. Time and time squared were entered as continuous fixed effects measured in days and then divided by 7 to assess weeks from baseline, allowing us to examine differences in change over time and account for variability in survey completion dates between participants. Data analyses were organized by hypothesis, as specified below.

One multilevel model was run with the *lme4* package (Version 1.1-38; [53]) with SUD severity as the dependent variable to test hypotheses 1a and 1b simultaneously. The model included 13 fixed effects including the within- and between-person variables for selected predictors (mindfulness, DBT skills, and emotion dysregulation), linear and quadratic time trends, and demographic and control covariates (e.g., age, sex, race/ethnicity, treatment arm, and baseline SUD severity). A second model with functional disability as the dependent variable was run with the same fixed effects to test hypothesis 3 and serve as a sensitivity analysis. All models included a random intercept for participant and assessed whether the inclusion of the random slopes for time (linear and quadratic) and other time-varying variables improved model fit (tested one by one) using a diagonal variance-covariance structure, which was used to reduce convergence problems.

Preliminary analyses indicated that treatment acceptability was negatively skewed, requiring reverse-scoring (total score+1) and a log transformation to achieve pseudo-normality. Usability was pseudo-normal when the mean score was used. For hypothesis 2a, time-varying moderators were disaggregated in the same way reported above to extract between-person and within-person variables (e.g., treatment acceptability, ease of use). Two multilevel models were run with SUD severity as the dependent variable and 11 fixed effects. Each model included the within-person moderator variable added as an interaction term with time to assess moderation (e.g., [predictor]*time, [predictor]*time^2^) producing 5 fixed effects, with the between-person moderator variable, age, sex, ethnicity, treatment arm, and baseline SUD severity added as covariates. For hypothesis 2b, these moderators were only available as person-level (Level 2) aggregates and were not time-varying (e.g., total hours and unique days spent on iDBT). After log-transformation due to positive skew, two multilevel models were run with SUD severity as the dependent variable and 10 fixed effects. The total hours and unique days were grand-mean-centered and entered as an interaction term with time to assess moderation (e.g., [predictor]*time, [predictor]*time^2^), producing 4 fixed effects, with the between-person moderator variable, age, sex, ethnicity, treatment arm, and baseline SUD severity added as covariates. For hypothesis 3, we repeated these analyses with functional disability as the dependent variable. For transparency, outputs from R are reproduced in Appendix in S1 Text.

All final models included the random intercept and any random slopes that were found to improve model fit using a diagonal variance-covariance structure. Across all multilevel models, restricted maximum likelihood (REML) estimation was used to evaluate all fixed effects, but all model comparisons were evaluated using maximum likelihood estimation with the *lmerTest* (Version 3.2-0; [54]) package, which uses Satterthwaite’s degrees of freedom method. An optimizer called *optimx* (Version 2025-4.9) set to the “nlminb” method (i.e., Nonlinear Minimization subject to Box Constraints) was used in all models. All analyses were two-tailed with an alpha of.05, and reported effects are unstandardized. Given the number of models (10 total) and fixed effects per model, we used a Benjamini-Hochberg correction on reported *p-*values to control for Type I error, as well as a Robust Effect Size Index (RESI; [55]) to aid interpretation of statistical findings. We also explored potential differences in results when using generalized lined mixed models (with package glmmTMB; Version 1.1.13) and negative binomial distributions.

## Results

### Preliminary within- and between-person correlations

As seen in Table 1, a few significant correlations between demographic variables and other variables were found at baseline, other than a positive relationship between age and SUD severity. Many other variables demonstrated anticipated univariate associations at baseline. Between-person SUD severity was positively associated with functional disability and emotion dysregulation as well as negatively associated with mindful awareness. Between-person functional disability was also positively associated with emotion dysregulation and negatively associated with mindful awareness. These relationships were consistent at the within-person level as well. Between-person emotion dysregulation was negatively associated with mindful awareness, and this extended to the within-person level as well. DBT skills were negatively associated with functional disability and emotion dysregulation at the within-person level only.

### Hypotheses 1: Within- and between-person associations with SUD severity

As seen in Table 2, SUD severity improved over the course of treatment, and people with higher SUD severity reported more change over time. Neither demographic characteristics (i.e., age, sex, or ethnicity) nor treatment arm were associated with treatment outcome. Supporting hypothesis 1a, within-person increases in emotion dysregulation were associated with increased SUD severity over the course of iDBT (i.e., they impeded improvements) over and above between-person variability. In contrast with hypothesis 1b, within-person mindfulness and DBT skills were not significantly associated with SUD severity over and above their between-person covariates. Moreover, none of the between-person effects were significant on their own.

**Table 2:** Final models incorporating all fixed and random effects of between- and within-person skills variables predicting substance dependence and functional disability.

	*Model 1: SUD Severity (N = 72, k = 254)*						*Model 2: Functional Disability (N = 72, k = 254)*					
	*b*	*SE*	*p*	*Adj. p*	*95% CI*	*RESI*	*b*	*SE*	*p*	*Adj. p*	*95% CI*	*RESI*
Intercept	7.20	.57	<.001	<.001	6.08, 8.32	1.54^c^	15.12	1.13	<.001	<.001	12.91, 17.33	1.61^c^
*Between-Person Variables*												
Age	.03	.02	.07	.14	-.002,.06	.19^a^	-.003	.03	.91	.91	-.06,.06	<.01
Sex (1 = female, 0 = male)	-.70	.39	.07	.14	-1.46,.05	.16^a^	-1.16	.78	.14	.25	-2.70,.37	.14^a^
Race (1 = White, 0 = other)	.05	.36	.88	.88	-.66,.77	<.01	-.14	.74	.85	.91	-1.59, 1.30	<.01
Treatment Arm	.25	.35	.49	.62	-.44,.93	<.01	-.13	.67	.85	.91	-1.45, 1.19	<.01
Weeks	**-.40**	**.08**	**<.001**	**<.001**	**-.57, -.24**	**.45** ^**c**^	-.35	.17	.047	.13	-.69, -.007	.16^a^
Weeks^2^	**.02**	**.01**	**.002**	**.006**	**.008,.03**	**.31** ^**b**^	.02	.01	.17	.26	-.008,.04	.08
Baseline DV Covariate	**.83**	**.06**	**<.001**	**<.001**	**.72,.94**	**1.68** ^**c**^	**.72**	**.04**	**<.001**	**<.001**	**.63,.81**	**1.60** ^**c**^
DBT Skills	.36	.47	.44	.62	-.56, 1.27	<.01	.48	.94	.61	.77	-1.37, 2.34	<.01
Emotion Dysregulation	.03	.02	.18	.28	-.01,.07	.08	.03	.05	.50	.70	-.06,.12	<.01
Mindfulness Skills	-.10	.31	.74	.86	-.71,.51	<.01	-1.50	.62	*.02*	*.07*	-2.71, -.28	.24^a^
*Within-Person Variables*												
DBT Skills	-.14	.69	.84	.88	-1.49, 1.21	<.01	-2.09	1.29	.11	.22	-4.61,.43	.15^a^
Emotion Dysregulation	**.08**	**.02**	**.002**	**.006**	**.03,.12**	**.35** ^**b**^	**.16**	**.05**	**.001**	**.005**	**.06,.26**	**.39** ^**b**^
Mindfulness Skills	.43	.32	.18	.28	-.20, 1.06	.04	-1.24	.67	.06	.14	-2.55,.07	.15^a^

*Note*: SUD = Substance Use Disorder. DBT = Dialectical Behavioural Therapy. *N* = Number of participants; *k* = number of observations. *Adj. p* represents the adjusted p-value after correction using the Benjamini-Hochberg method. Bold added to highlight significant effects after correction. A random intercept and all significant random slopes were included in each model using an uncorrelated diagonal setting for random effect variances. RESI = Robust Effect Size Index, interpretation [55]: ^a^Small-medium; ^b^Medium-large; ^c^Large.

### Hypotheses 2: Moderation of engagement between time and SUD severity

For hypothesis 2a, only one significant interaction between usability and time was found, whereby higher within-person usability scores (i.e., lower ratings of usability) were associated with reduced changes in SUD severity. However, although this relationship produced a small-medium effect size, it was not significant after adjusting for multiple tests. For hypothesis 2b, we did not find any significant interactions for either behavioral engagement variable (i.e., hours or unique days spent on iDBT), suggesting that increased time spent on iDBT did not enhance or diminish the treatment effects found for SUD severity, though these variables did produce small-medium effect sizes.

### Hypotheses 3: Replication of findings with functional disability

As seen in Table 2, functional disability did not improve over the course of treatment, but people with higher disability still reported greater changes over time. Neither demographic characteristics nor treatment arm were associated with treatment outcome. Supporting hypothesis 3, we replicated a SUD severity effect demonstrating that within-person increases in emotion dysregulation were associated with increased functional disability (i.e., they impeded improvements) over and above between-person variability. Also, consistent with SUD severity, within-person mindfulness and DBT skills were not significantly associated with functional disability over and above their between-person effects. Higher between-person mindfulness levels were associated with lower functional disability, but this effect was not significant after corrections for multiple tests.

In contrast to hypothesis 3 (see Table 3), there were no consistent within-person moderation effects when comparing functional disability to SUD severity. One significant between-person main effect emerged whereby lower ratings of usability (i.e., higher scores on average) were associated with greater functional disability scores over the course of treatment. However, although this relationship produced a small-medium effect size, it was not significant after adjusting for multiple tests.

**Table 3:** Effects of time-varying (within-person) and between-person moderation on substance dependence and functional disability as treatment outcomes.

	*DV: SUD Severity*					*DV: Functional Disability*				
	*b*	*SE*	*p*	*Adj. p*	*RESI*	*b*	*SE*	*p*	*Adj. p*	*RESI*
*Treatment Acceptability, total score (N = 63, k = 149) [reversed and log transformed]*										
Time × Moderator (WP)	1.53	1.06	.15	.28	<.01	1.08	2.27	.64	.91	<.01
Time^2^ × Moderator (WP)	-.08	.06	.20	.30	<.01	-.06	.13	.67	.91	<.01
WP Main Effect	-6.49	3.98	.11	.23	<.01	-4.52	8.49	.60	.91	<.01
BP Main Effect	.25	.44	.57	.63	<.01	.77	.93	.41	.91	<.01
*mHealth Usability, average score (N = 63, k = 146) [not transformed]*										
Time × Moderator (WP)	**3.22**	**1.54**	**.04**	**.09**	**.18** ^**a**^	2.00	3.24	.54	.89	<.01
Time^2^ × Moderator (WP)	**-.20**	**.09**	**.03**	**.09**	**.20** ^**a**^	-.10	.20	.59	.89	<.01
WP Main Effect	**-11.42**	**5.79**	**.05**	**.09**	**.16** ^**a**^	-7.41	12.22	.54	.89	<.01
BP Main Effect	-.01	.48	.98	.98	<.01	**2.24**	**.99**	**.02**	**.08**	**.24** ^**a**^
*Engagement Hours (N = 72, k = 261) [log transformed]*										
Time × BP Moderator	**.09**	**.06**	**.12**	**.17**	**.15** ^**a**^	-.12	.12	.33	.77	<.01
Time^2^ × BP Moderator	**-.007**	**.004**	**.12**	**.17**	**.15** ^**a**^	.004	.009	.66	.89	<.01
BP Main Effect	.001	.18	.99	.99	<.01	.09	.36	.81	.89	<.01
*Unique Login Days (N = 72, k = 261) [log transformed]*										
Time × BP Moderator	.05	.06	.38	.46	.06	-.11	.13	.38	.84	.04
Time^2^ × BP Moderator	**-.005**	**.005**	**.22**	**.35**	**.14** ^**a**^	.002	.01	.80	.96	<.01
BP Main Effect	-.01	.18	.96	.96	<.01	-.02	.37	.96	.96	<.01

Note: SUD = Substance use disorder. DV = Dependent Variable; WP = Within-persons; BP = Between-persons. * *p* < .05, ** p < .01, *** *p* < .001. WP interactions and main effects added for time-varying variables measured at follow-up (greater data missing because measures were administered post-baseline only). BP interactions and main effects were added for engagement variables because they were not time-varying. All models included the following 7 covariates (not shown above): age, sex, ethnicity, treatment arm, time (linear and quadratic), and baseline dependent variable. *Adj. p* represents the adjusted p-value after correction using the Benjamini-Hochberg method. Lower usability scores are considered better ratings. Bold type was used to highlight variables that achieved at least a small effect size. RESI = Robust Effect Size Index, interpretation [55]: ^a^Small-medium; ^b^Medium-large; ^c^Large.

### Sensitivity analyses

Given the pseudo-normal nature of our SUD severity and functional disability variables (e.g., scores were positive, continuous variables but over dispersed with variances greater than their means), we ran exploratory analyses using generalized linear mixed models specifying a negative binomial distribution to determine the robustness of our findings. As reported in Table A in S1 Text, after all significant random slopes were entered and the final converged model was evaluated, we found the same relationships between higher within-person emotion dysregulation and greater SUD severity and functional disability. The only additional effect to emerge was a positive association between age and SUD severity.

In regard to moderation effects, the generalized linear mixed models (see Table B in S1 Text) performed similarly to the models reported above. After controlling for multiple tests, there were no significant within- or between-person moderation effects for SUD severity. The closest moderation effects to achieve significance for SUD severity again involved usability. For functional disability, there emerged a significant between-persons main effect with usability, whereby higher scores (i.e., lower usability ratings) were associated with higher functional disability through iDBT. No other within- or between-person moderation effects were found.

## Discussion

The aim of the current study was to examine potential mechanisms of treatment outcome in individuals with SUDs who received iDBT, as well as the potential moderating effect of engagement. Importantly, however, all associations observed in this study are correlational in nature, and no causal inferences can be drawn regarding mechanisms or treatment effects. Considering the literature on both traditional and internet-delivered DBT formats, we examined whether the between- and within-person changes in DBT skills, mindfulness, and emotion dysregulation were associated with treatment response for individuals with SUDs. Moreover, we considered how time-varying indices of cognitive engagement (e.g., treatment acceptability and usability) as well as between-person behavioural engagement (e.g., hours/days spent on iDBT) are associated with enhanced or diminished treatment effects. This study builds on prior findings by offering a more nuanced understanding of how psychological constructs fluctuate and interact during iDBT, and DBT more broadly.

Results partially supported our first hypothesis. Within-persons, higher emotion dysregulation (i.e., difficulties in emotion regulation) at each timepoint was associated with diminished SUD severity reductions in iDBT, and these findings replicated with functional disability as an outcome. These findings support theoretical frameworks suggesting that impaired emotion regulation is associated with the development and maintenance of SUDs [6,8] and that improvements predict change in standard DBT for a range of mental health problems, such as depression, anxiety, and self-harm [30,31]. The present study was able to extend these findings to individuals with SUDs undergoing iDBT, suggesting that changes in emotion (dys)regulation are also likely important for favorable outcomes in this context. While we did not find within-person effects for mindfulness or DBT skills, these findings add to previous research examining these variables as potential mechanisms in standard DBT [28,29,56]. The discrepancy in findings could be due to the different format of treatment, the sample recruited, or the dose (neither of these previous studies had a focus on SUDs nor iDBT). It is possible that mindfulness and DBT skill acquisition requires time to integrate into daily life, and its effects may not be immediately observable within the 12-week timeframe of the current study; it is also challenging to confirm use of skills asynchronously. More broadly, there is a lack of research on the mechanisms of DBT treatment, making it difficult to compare findings across studies. More studies are needed to understand how people improve during DBT, especially those that consider multilevel contributions of within- and between-person variance.

We found a small effect size relationship whereby individuals who had higher (i.e., between-person) levels of dispositional mindfulness reported lower symptoms of functional disability. Thus, mindfulness skills may be associated with certain treatment outcomes in individuals with SUDs undergoing iDBT. This effect mirrors several results found in the literature. For example, Mitchell et al. [57] found that between-person changes in mindfulness were associated with reductions in BPD symptom severity, depression, and distress in a sample of BPD individuals undergoing group DBT. In other studies, it was reported that between-person mindfulness increased over the course of standard DBT and iDBT but was not necessarily a predictor of treatment outcome [30,58,59]. Dispositional mindfulness can be seen as a protective factor because it has been associated with greater self-awareness and fewer impulsive behaviors [60], factors which may be related to greater behavioral control in those with SUDs. Our findings suggest that more stable, trait-like individual differences in mindfulness may have a stronger influence on long-term disability than week-to-week fluctuations [61]. Additionally, greater mindfulness abilities gained in treatment have been linked to greater gains in emotion regulation ability in at least two studies (one on mindfulness-based stress reduction [62] and another on standard DBT [56]), suggesting that it may be worth continued study.

With regards to hypothesized moderation of treatment effects, our hypotheses were generally not supported. While we did find some evidence of a potential moderation effect on SUD severity for usability and engagement hours in the form of a small-medium effect size, results remained non-significant and did not replicate to functional disability. For our second outcome, only higher between-person ratings of usability indicated potential association with lower functional disability. Given that engagement with iDBT likely reflects an array of cognitive and motivational factors beyond just behavioural forms of engagement (e.g., time spent on iDBT) we wanted to assess whether different types of engagement could moderate its treatment response. Higher acceptability and usability may shape how often participants attend to, interpret, and integrate intervention material, which could strengthen the association between time in treatment and symptom change. The fact that we found differences for different types of engagement variables may reflect important distinctions between these constructs in digital interventions. Behavioral metrics quantify usage but do not indicate the quality of engagement, intentionality, or depth of processing. In asynchronous iDBT, participants may spend comparable amounts of time on the platform while differing substantially in how actively they reflect on, practice, or generalize skills. From a research perspective, they underscore the need to assess engagement multidimensionally when evaluating digital treatment processes. There are some findings from the literature that align with our work. Wilks et al. [63] reported that participants who found the first module less useful and encountered technological difficulties were more likely to dropout from iDBT for SUDs, which aligns with our findings on between-person usability ratings. Another study examined the relationship between engagement on iDBT and disordered eating symptoms; however, the authors reported that neither module completion nor diary card entries were moderators of change [38]. A separate study on the same iDBT app for disordered eating found that acceptability could be cultivated through performance expectancy (i.e., perceiving that the app would be beneficial) and facilitation (i.e., app infrastructure and support; [64]). Collectively, these findings suggest continued research to investigate the potential associations between different forms of engagement and treatment outcomes in iDBT.

## Limitations and future directions

Despite the strengths of this study, several limitations should be considered when interpreting the findings. First, while multilevel modeling is robust to modest violations of normality, several of the variables in this study were only approximately normally distributed even after transformation. This pseudo-normality may have increased residual errors, affecting the estimation of fixed effects, variance components, and interaction terms. We included sensitivity analyses with generalized linear mixed models to report potential differences. Second, this secondary analysis of a single-blind RCT was not originally powered to test moderation or multilevel mediation. The modest number of participants limited power for detecting between-person (Level-2) moderation effects and other small or complex interaction effects. Similarly, the use of multiple hypothesis tests increases susceptibility to both Type I and Type II error, despite correction for multiple comparisons. Third, the sample consisted of treatment-seeking individuals who voluntarily enrolled in a digital intervention, which may reflect a subset of people with higher digital literacy, motivation for change, or comfort with self-guided tools. As a result, engagement levels and treatment responses observed in this study may overestimate what would be expected in broader SUD populations, particularly among individuals with lower motivation, limited access to or familiarity with, digital technologies, or more severe psychiatric and social complexity. From a research perspective, these findings may therefore reflect the functioning of digital interventions under conditions of optimal fit rather than typical uptake. This underscores the need for futures studies that examine engagement and outcomes in more heterogeneous samples, including individuals recruited from clinical or community-based pathways.

Fourth, while we found some support for prediction of treatment outcome and some evidence of moderation, it is important to note that these relationships are still correlational. While we may hypothesize that increased skill acquisition and engagement lead to reduced SUD severity and disability, it is also plausible that reductions in symptoms facilitate greater skill learning and engagement. Therefore, causation or direction of effects cannot be assumed. Finally, the study duration of 12 weeks may have been insufficient to observe longer-term changes in skill acquisition and or symptom changes. Longer follow-up periods may be needed to assess the stability of these effects and the impact of skill acquisition and engagement on long-term treatment outcomes.

## Conclusion

This study underscores the importance of emotion regulation and mindfulness in relation to treatment outcomes for individuals with SUDs undergoing iDBT. Moreover, both cognitive and behavioural engagement in the form of positive treatment perceptions and time spent on iDBT may enhance outcomes. Future research should consider the benefit of measuring these skill-building outcomes to illuminate the possible mechanisms of treatment underlying DBT in various formats as well as predictors of improved treatment response. While our findings are preliminary, there are few studies involving iDBT and therefore these results may help inform future research within different target populations and formats of DBT. Overall, iDBT for SUDs was able to enhance mindfulness and emotion regulation skills much like standard DBT and may be helpful for other diverse populations, enhancing its potential for scalability. Larger-scale investigations with longer follow up periods will meaningfully extend this foundational research.

## Supporting information

S1 Text Sensitivity analyses and statistical outputs of all models presented in manuscript. This file includes multilevel model results not reported in the main manuscript (Tables A and B), and statistical outputs of models presented in the manuscript (Appendix).
(DOCX)
