# Supplementary material for: Multilevel associations between skills use, engagement, and treatment outcome in self-guided internet-delivered dialectical behavior therapy for substance use disorders
Source: PLOS Ment Health. 2026 Mar 24;3(3):e0000564. doi: 10.1371/journal.pmen.0000564 (PMC13012472; doi:10.1371/journal.pmen.0000564)
Supplement: S1 Text — This file includes multilevel model results not reported in the main manuscript (Tables A and B), and statistical outputs of models presented in the manuscript (Appendix). (DOCX) [file pmen.0000564.s001.docx]

**Appendix S1 Text**

Table A S1 Text. Sensitivity analyses with fixed and random effects of between-
and within-person skills use variables predicting depression and anxiety.

|  | *Model 3: Depression and Anxiety* *(N = 72, k = 254)* | | | |
| --- | --- | --- | --- | --- |
|  | *b* | *SE* | *t* | *95% CI* |
| Intercept | 19.82 | 1.27 | 15.65*** | 17.34, 22.31 |
| *Between-Person Variables* |  |  |  |  |
| Age | .03 | .03 | 1.00 | -.03, .10 |
| Sex (1 = female, 0 = male) | -2.98 | .89 | -3.34** | -4.74, -1.23 |
| Race (1 = White, 0 = other) | -.30 | .86 | -.35 | -1.98, 1.38 |
| Treatment Arm | .68 | .77 | .88 | -.84, 2.20 |
| Weeks | -.58 | .20 | -2.84** | -.98, -.18 |
| Weeks^2^ | .02 | .02 | 1.61 | -.005, .05 |
| Baseline DV Covariate | .68 | .05 | 13.51*** | .58, .78 |
| DBT Skills | .75 | 1.06 | .71 | -1.33, 2.83 |
| Emotion Dysregulation | .08 | .05 | 1.45 | -.03, .18 |
| Mindfulness Skills | -2.36 | .70 | -3.40** | -3.73, -1.00 |
| *Within-Person Variables* |  |  |  |  |
| DBT Skills | -1.69 | 1.40 | -1.21 | -4.25, 1.05 |
| Emotion Dysregulation | .21 | .06 | 3.72*** | .10, .32 |
| Mindfulness Skills | .06 | 1.00 | .06 | -1.90, .78 |

*Note*: * *p* < .05, *** p* < .01, *** *p* < .001. DBT = Dialectical Behavioural Therapy. *N* = Number of participants; *k* = number of observations. A random intercept and all significant random slopes were included in each model using an uncorrelated diagonal setting for random effect variances.

Table B S1 Text. Sensitivity analyses with effects of time-varying (within-person) and between-person moderation of depression and anxiety as a treatment outcome.

|  | *DV: Depression and Anxiety* | | | |
| --- | --- | --- | --- | --- |
|  | *b* | *SE* | *t* | *p* |
| *Treatment Acceptability* |  |  |  |  |
| Time × Moderator (WP) | .35 | .41 | .84 | .40 |
| Time^2^ × Moderator (WP) | -.02 | .03 | -.85 | .40 |
| BP Covariate Main Effect | -.17 | .17 | -1.01 | .32 |
| *Ease of Use* |  |  |  |  |
| Time × Moderator (WP) | -2.00 | 1.69 | -1.19 | .24 |
| Time^2^ × Moderator (WP) | .15 | .10 | 1.51 | .13 |
| BP Covariate Main Effect | 1.34 | .94 | 1.43 | .16 |
| *Interface and Satisfaction* |  |  |  |  |
| Time × Moderator (WP) | 2.70 | 1.61 | 1.67 | .10 |
| Time^2^ × Moderator (WP) | -.13 | .09 | -1.44 | .16 |
| BP Covariate Main Effect | .71 | .90 | .79 | .43 |
| *Usefulness* |  |  |  |  |
| Time × Moderator (WP) | -.81 | 2.03 | -.40 | .69 |
| Time^2^ × Moderator (WP) | .05 | .12 | .43 | .67 |
| BP Covariate Main Effect | -.44 | 1.05 | .42 | .68 |
| *Engagement Hours (log)* |  |  |  |  |
| Time × BP Moderator | -.31 | .15 | -2.07 | .04 |
| Time^2^ × BP Moderator | .02 | .01 | 1.68 | .09 |
| *Unique Days (log)* |  |  |  |  |
| Time × BP Moderator | -.39 | .16 | -2.54 | .01* |
| Time^2^ × BP Moderator | .03 | .01 | 2.26 | .02* |

Note: DV = Dependent Variable; WP = Within-persons; BP = Between-persons. * *p* < .05, *** p* < .01, *** *p* < .001. Within-person interactions for time-varying variables were used due to multiple assessments over follow-up (N=63; 147 observations). Between-person engagement variables were used for engagement variables (N=72; 254 observations) because they represented total hours and unique days over the course of the study that were not time-varying. For ease of use, interface and satisfaction, and usefulness, lower ratings are considered more favorable.
